# How metformin affects various malignancies by means of microRNAs: a brief review

**DOI:** 10.1186/s12935-021-01921-z

**Published:** 2021-04-13

**Authors:** Nahid Alimoradi, Negar Firouzabadi, Reihaneh Fatehi

**Affiliations:** Department of Pharmacology and Toxicology, School of Pharmacy, Shiraz University of Medical Sciences, Shiraz, Iran

**Keywords:** Metformin, MicroRNAs, Cancer, AMPK, Biomarker, Mechanism of action

## Abstract

Metformin known as the first-line orally prescribed drug for lowering blood glucose in type II diabetes (T2DM) has recently found various therapeutic applications including in cancer. Metformin has been studied for its influences in prevention and treatment of cancer through multiple mechanisms such as microRNA (miR) regulation. Alteration in the expression of miRs by metformin may play an important role in the treatment of various cancers. MiRs are single-stranded RNAs that are involved in gene regulation. By binding to the 3′UTR of target mRNAs, miRs influence protein levels. Irregularities in the expression of miRs that control the expression of oncogenes and tumor suppressor genes are associated with the onset and progression of cancer. Metformin may possess an effect on tumor prevention and progression by modifying miR expression and downstream pathways. Here, we summarize the effect of metformin on different types of cancer by regulating the expression of various miRs and the associated downstream molecules.

## Introduction

Metformin is a potent glucose-lowering agent with a biguanide structure that is effective in the treatment of type II diabetes (T2DM). Metformin adjusts cellular energy metabolism through inducing AMP-activated protein kinase (AMPK), an important metabolic sensor that is activated under cellular stress conditions and limits energy consumption by affecting ATP production and consumption [1, 2].

There is ample evidence that metformin can reduce the risk of various types of malignancies [3, 4]. However, there are still unresolved questions about the accurate mechanism of metformin in the treatment and prevention of cancer. One of the most well-known mechanisms of metformin as an anticancer drug is linked to the inhibition of the mammalian target of rapamycin complex 1 (mTORC1) through AMPK activation, directly and indirectly [4]. Furthermore, reported studies have shown that the effects of metformin on different type of cancers may be mediated via AMPK-dependent and AMPK-independent mechanisms and through regulation of the mitogen-activated protein kinase (MAPK), nuclear factor kappa B (NF-ĸB), protein kinase B [5] and mTOR signaling pathways [3, 6, 7].

**Fig. 1:**
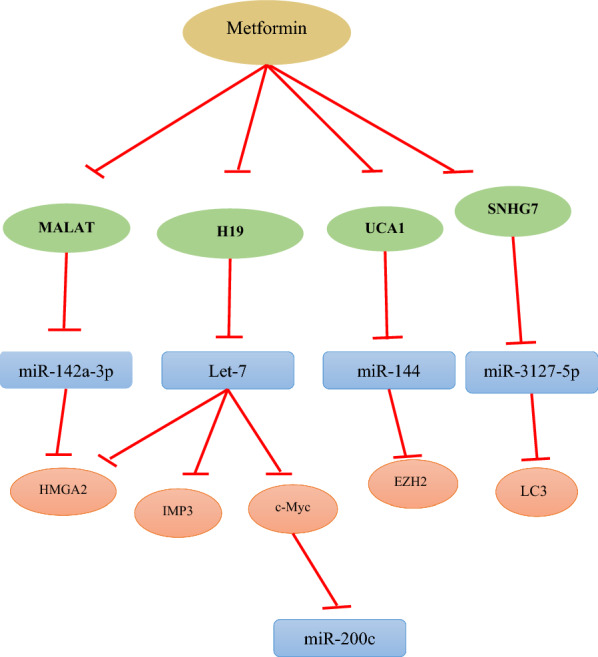
Regulation of various miRs through lncRNA inhibition and their associated signaling molecules by means of metformin

MicroRNAs (miRs) are small single-stranded and noncoding RNAs that are approximately 22 base pair in length [8]. MiRs regulate the expression of genes post-transcriptionally by binding to the untranslated region 3′ (3′UTR) of their messenger RNA (mRNA). Coupling of miRs to their target mRNA sequences leads to cleavage or repression of their translations [9, 10]. The discovery of new molecular mechanisms suggests that miRs can play a fundamental role in prognosis and diagnosis of cancer. MiRs act as important mediators in most cellular processes. Therefore, knowledge of the effective miRs and their mechanisms of action is essential for a more accurate conception and management of cancer [11]. Here, we discuss the role of metformin in regulating several signaling pathways involved in different types of cancer, which are associated with the regulation of numerous miRs, as shown in Table 1 and Fig. 1.

**Table 1:** Examples of miRNAs modulated by metformin in cancers and their putative mRNA targets

Cancer	miRNAs	Drug’s effect on miRNAs	Putative mRNA(s) targets	References
Breast cancer	miR-27b miR-26a miR-21-5p miR-21 miR-155 miR-708 miR-200c	Up Up Down Up Down Down Up	ENPP1 PTEN, MCL-1, EZH2, E2F3 & MTDH Sestrin-1 & CAB39L Foxo-1 Foxo-3 CD47 AKT2 and Bcl-2	[14] [23] [28] [29] [29] [35] [37]
Cervical cancer	miR-142-3p	Up	HMGA2	[40]
Cholangiocarcinoma	miR-302	Up	Rb, cyclin D1& Cdk4	[48]
Colorectal cancer	miR-21 miR-200 family miR-34a	Down Up Up	Spry2 ZEB SNAIL1	[54] [61] [61]
Endometrial Hyperplasia	let-7 miR-144	Up Up	c-Myc, HMGA2 & Imp3 EZH2	[47] [85]
Gallbladder cancer	miR-675-5p	Up	–	[89]
Gastric cancer	miR-15a miR-128 miR-192 miR-194	Up Up Up Up	Bmi-1 Bmi-1 Bmi-1 Bmi-1	[96] [96] [96] [96]
Hypo pharyngeal Carcinoma	miR-21-5p	Down	PDCD4	[101]
Non-small-cell lung Cancer	miR-7 miR-381 miR-107	Up Up Down	Bcl-2 YAP Eomes	[107] [112] [115]
Osteosarcoma	miR-570-3p	Up	LCMR1 and ATG12	[119]
Ovarian cancer	let-7 miR-3127-5p	Up Up	c-Myc, HMGA2 & Imp3 LC3	[47] [123]
Pancreatic cancer	miR-150 miR-210-5p miR-221 miR-200a-3p	Up Up Down Down	MUC4 PFKFB2 P27 –	[73] [136] [141] [149]
Prostate cancer	miR-708	Up	NNAT	[150]
Renal cell carcinoma	miR-21 miR-34a	Down Up	PTEN cyclin D1	[155] [159]
Skin cancer	miR-21 miR-192-5p miR-584-3p	Down Up Up	PTEN EFEMP1 SCAMP3	[166] [175] [175]

## Breast cancer

### MiR-27b

Expression of miR-27b is among the various miRs that are influenced by metformin. MiR-27b that is a tissue/cellular miR, does not have a constant behavior in cancer development and its action differs from type to type of cancer. While it plays an oncogenic role in some types of breast cancer, it has a tumor-suppressor role in luminal-type breast cancer and prevents side-population cell (SP fraction) generation [12–14]. Ectonucleotide pyrophosphatase/phosphodiesterase family member 1 (ENPP1), that is negatively modulated by metformin, is up-regulated in this type of breast cancer. It was found that 3′UTR of ENPP1 has a binding site for miR-27b and is a direct target of miR-27b [14, 15]. ENPP1 has the ability to induce SP fraction generation which is involved in the process of drug resistance and tumor genesis by altering cell surface efflux pumps such as ATP-binding cassette super-family G member 2 (ABCG2) and 26S proteasome activity [14, 16]. Metformin reduces SP fraction in breast cancer cells by up-regulating miR-27b [14].

### MiR-26a

Decreased expression of circulating miR-26 in several cancers such as breast cancer indicates the tumor-suppressing role of miR-26 [17–22]. On the other hand, increased expression of miR-26a by metformin, has shown to significantly inhibit cell viability in breast cancer cells. MiR-26a directly targets several anti-apoptotic proteins, such as E2F Transcription Factor 3 (E2F3), myeloid cell leukaemia-1 (Mcl-1), metadherin (MTDH), enhancer of zeste homolog 2 (EZH2), and phosphatase and tensin homolog (PTEN) which are up-regulated in breast cancer. Over-expression of miR-26a by metformin down-regulates these targets in breast cancer cells [23–25].

### MiR-21 and miR-155

MiR-21 is one of the prominent up-regulated oncomiRs in many types of cancers such as breast cancer cells [26, 27]. Metformin inhibits proliferation and metastasis via down-regulation of miR-21-5p in breast cancer cell lines. Evidences have also shown a decrease in miR-21-5p levels in serum of patients with a history of breast cancer who were treated with metformin. MiR-21-5P suppresses the expression of calcium-binding protein 39-like (CAB39L) and Sestrin-1, the activators of AMPK, thus causing decreasing AMPK activation and increasing mTOR signaling. These results suggest that metformin activates AMPK and inhibits mTOR in breast cancer by inhibiting miR-21-5p [28].

The results of another study which has been carried out in various types of breast cancer cell lines with different hormonal backgrounds including MCF-7, MDA-MB-231, and T47D indicated that metformin treatment significantly inhibits breast cancer metastasis, through down-regulation of two oncogenic circulating miRs, miR-21 and miR-155. Aberrant expression levels of these two miRs are linked to invasive and metastatic properties of cancerous cells [29, 30]. MiR-21 and miR-155, both target forkhead box class O 3a (FOXO3a) in breast cancer which acts as a tumor suppressor [31, 32]. It has been shown that metformin is associated with AMPK and FOXO3a activation in MCF-7 breast cancer cells [33].

### MiR-708

A study on breast cancer stem cells conveyed that metformin could indirectly affect immune-related genes such as cluster of differentiation 47 (CD47) that are involved in resistance to chemotherapy. Indeed, metformin treatment could cause the restoration of miR-708 that is a cellular/tissue miR and decrease the expression level of CD47 which consequently results in chemo-sensitivity in breast cancer. CD47 is over-expressed in various cancers and provides inhibitory actions when it binds to the signal regulatory protein alpha (SIRPα) receptor, which is expressed on macrophages and prevents phagocytosis [34, 35].

### MiR-200c

MiR-200c is a circulating miR with the potential as a biomarker for breast cancer prognosis [36]. Metformin inhibits metastatic process and proliferation in breast cancer cells by reducing the expression of cellular Myb (c-Myc). c-Myc can bind to the miR-200c promoter leading to its inhibition. Metformin inhibits c-Myc causing miR-200c up-regulation. Up-regulation of miR-200c is associated with a decrease in the amount of AKT2 and B-cell lymphoma 2 (Bcl-2) which are directly targeted by miR-200c [37].

## Cervical cancer

### MiR-142

MiR-142-3p, a tissue/cellular miR with a tumor suppressive role, which is down-regulated in several types of cancer may serve as a biomarker for diagnosis and prognosis of specific malignancies [38, 39]. MiR-142 directly targets high-mobility group protein A2 (HMGA2) which is an oncogene. By this means, it inhibits the proliferation and invasion of cervical cancer cells [40]. Based on previous reports, HMGA2 plays a role in epithelial–mesenchymal transition (EMT) and is over-expressed in several cancers and is responsible for the invasive and metastatic behaviors of cancer cells [41–43]. Besides, metastasis-associated lung adenocarcinoma transcript 1 (MALAT1) which is a long noncoding RNA (lncRNA), is elevated in various cancer types such as osteosarcoma, breast, prostate and cervical cancer [40, 44–46]. MALAT1 binds to miR-142–3p like a sponge and prevents its inhibitory effect on HMGA2. Metformin may inhibit MALAT1 expression by affecting DNA methylation of the MALAT1 promoter. Inhibition of the formation of MALAT1/miR-142-3p complex disrupts HMGA2 expression, and thus the free form of miR-142-3p is increased [40, 47]. In this manner, metformin exerts its anticancer actions by targeting miR-142 indirectly.

## Cholangiocarcinoma

### MiR-302

In a study conducted by Fujimori et al., the anti-proliferative effects of metformin on the cholangiocarcinoma cell line and tumor tissue and the expression of a number of miRs were analyzed. It was observed that along with the inhibitory effects of metformin on the proliferation of these cells and cell cycle (G0 to G1), the expression of miR-302b,c increased and cell cycle regulator proteins such as retinoblastoma (Rb), cyclin D1, and Cdk4 decreased [48, 49]. Complexes of cyclin-dependent kinases (CDKs) such as CDK4 and CDK6 with cyclin D1 are required for G1 phase progression [50]. Various studies have shown that metformin has the ability to down regulate cyclin D1 in various cancer cell lines [51–53]. It is possible that metformin mediates cell cycle regulators (cyclin D1, CDK4, and phosphorylated Rb) by over-expressing miR-302 in cholangiocarcinoma.

## Colorectal cancer

### MiR-21

A study regarding treatment of colon cancer cells with metformin indicated that metformin increases the expression level of Sprouty2 (Spry2), and PTEN by inhibiting the expression of miR-21 which in turn represses cell growth [54, 55]. Over-expression of circulating miR-21 is related to disease progression in colorectal cancer [56]. Studies have shown that up-regulation of miR-21 is associated with down-regulation of Spry2 in certain types of cancer. Thus, Spry2 could be described as a direct target of miR-21 [54, 55]. Spry2 is identified as a negative regulator of receptor tyrosine kinases (RTKs) which inhibit extracellular signal-regulated kinase (ERK) /MAP kinase pathways [55, 57, 58]. In addition, Spry2 increases the level of PTEN that in turn alleviates the activity of Akt leading to inhibition of mTORC1 [55, 59, 60].

### MiR-34a and miR-200 family

Metformin inhibits the over-expression of classical EMT markers such as SNAIL and zinc finger E-box binding homeobox (ZEB) by potentiating the expression of miR-34a and miR-200 to prevent colorectal cancer progression and/or tumor recurrence [61]. Metformin also exhibits its anti-EMT effects by altering the ratio of E-cadherin, an epithelial marker, to vimentin, a mesenchymal marker [61]. SNAIL and ZEB repress E-cadherin transcription in different ways [62, 63]. Studies on colorectal cancer suggested that down-regulation of circulating miR-34 and miR-200 family contributes to EMT and metastatic behavior [64–67]. Surprisingly, miR-34a was decreased in metformin treated cells. This action may be related to the inducing effects of metformin on Sirtuin-1 which is involved in the p53-miR-34a-Sirtuin axis and the generation of reactive oxygen species [68]. The results of studies indicate that metformin-induced p53 protein level in wild type p53 cancer cells lead to up-regulation of miR-34a. Induction of miR-34a leads to down-regulation of Sirtuin-1. Sirtuin-1 exhibits oncogenic properties in wild-type p53 cancer cells while acting as a tumor suppressor in mutant p53 cancer cells [76]. These results suggest that the effect of metformin in cancer cells depends on the status of p53. Metformin reduces Sirtuin1 protein levels only in wild type p53 cancer cells but does not affect the Sirtuin1 protein level in mutant forms of p53 [69, 70]. A well-established in-vitro senescence model treated with metformin has shown that metformin is able to accelerate the onset of stress-induced senescence (SIS), as well as lowering its threshold. It was also found that metformin lowers the threshold of SIS by simultaneous regulation of miR-205 and miR-200 family leading to inhibition of the expression of ZEB1 and ZEB2 transcription factors [71].

## Endometrial hyperplasia

### Let-7

Metformin suppresses cell migration and invasion by increasing let-7 expression which is down-regulated in endometrial cancer cells. Let-7 is a well-known tumor suppressor circulating miR that targets mRNAs of oncogenic genes such as c-Myc, HMGA2 and insulin-like growth factor 2 mRNA-binding protein 3 (Imp3) [47, 72]. It is assumed that metformin affects let-7 expression by different mechanisms altering H19/let-7 axis, Lin28/let-7 axis and AMPK phosphorylation. Metformin may also act in a DICER1‐dependent manner and apply its tumor suppressing effects [47, 73–76]. MiRs maturation takes place in several stags, from the nucleus to the cytoplasm, by several enzymes among which DICER, an RNase III nuclease, is the last enzyme [77]. H19, a lncRNA, which is elevated in various cancer types, binds to let-7 like a sponge and inhibits its function [78]. Additionally, Lin28, an RNA-binding protein that induces the pluripotency of adult human fibroblast cells and suppresses the production of let-7, is over-expressed in advanced cancers [79, 80]. Intriguingly, H19 and LIN28 expression is also down-regulated by let-7 [81]. Lin28 prevents the effect of DICER on let-7 by binding to the pre-let-7 terminal loop and induces pre-let-7 terminal uridylation by TUTase 4 (TUT4), a uridylyl transferase. In this way, DICER cannot remove this uridylated domain and convert pre-let-7 to the mature form of let-7, thus it negatively regulates let-7 expression [82, 83]. Lin28 indirectly increases H19 expression through inhibition of let-7. In contrast, H19 indirectly increases Lin28 levels by binding to let-7 and depresses its free form. As a result, both inhibit let-7 function through a double-negative feedback loop mechanism [81]. Metformin enhances methylation of H19 promoter and facilitates S-adenosyl-methionine (SAM)-dependent methyltransferase by activating S-adenosyl homocysteine hydrolase (SAHH), which is itself inactivated by binding to H19 [47, 76, 84]. Hence, metformin induces over-expression of let-7 by inhibiting H19 and LIN28 in endometrial cancer.

### MiR-144

Metformin exerts its antitumor effects in endometrial hyperplasia by triggering the expression of miR-144, which is a cellular miR, and active caspase 3 through decreasing the expression level of urothelial cancer-associated 1 (UCA1), an oncogenic lnc RNA [85]. Studies have indicated that decreased miR-144 expression is closely associated with tumor cell proliferation and invasion in various cancers such as endometrial hyperplasia [86]. Following an increase in miR-144, in metformin-treated endometrial cancer cell lines, transforming growth factor-β (TGF-β) and p-AKT levels also decrease [85]. Disrupted TGF-β signaling is associated with endometrial cancer. UCA1 up-regulation demonstrates its tumorigenic role by inducing the expression of TGF-β1. Metformin prevents TGF-β1 induction by inhibiting UCA1 [87, 88]. Evidence has shown that miR-144 targets 3′UTR of Enhancer of zeste homolog 2 (EZH2), a transcriptional repressor, suppresses the expression of EZH2 and inhibits proliferation, migration and invasion in human endometrial cancer cell lines [86].

## Gallbladder cancer

### MiR-675-5p

A study performed on human gallbladder cancer cell line, revealed that metformin could suppress cell proliferation and tumor growth via regulating various miR expression profiles. In this study, 35 miRs were identified and their expression levels were significantly changed after metformin treatment. Among these miRs, miR-675-5p, a cellular miR, was reported to be correlated with the anti-tumor effect of metformin. Part of the growth inhibitory effect of metformin could be due to miR-675-5p expression as metformin increased its expression in these cells as well [89]. MiR-675-5p has been characterized as a tumor suppressor in several human cancers. Decreased expression of miR-675-5p in various cancers such as gallbladder cancer, led to enhanced cell growth, proliferation, colony formation, invasion, and migration [89–91].

## Gastric cancer

### MiR-15a, miR-128, miR-192 and miR-194

B-lymphoma moloney murine leukemia virus insertion region-1 (Bmi-1) as an oncogene, is involved in self-renewal and differentiation of stem cells, tumor genesis and metastasis [92, 93]. Bmi-1 over-expression was reported to be closely associated with different malignancies [94, 95]. Bmi-1 is the direct target of miR-15a, miR-128, miR-192 and miR-194, the tumor suppressor miRs that are up-regulated in human gastric carcinoma cells and tissue samples treated with metformin [96]. Circulating miR-192 is known as a diagnostic and prognostic biomarker in gastric cancer [97]. Metformin as an AMPK activator, inhibits cell migration, invasion and cancer progression by up-regulating these miRs [98, 99]. Activation of AMPK leads to over-expression of miR-15a, miR-128, miR-192 and miR-194 thus reducing Bmi-1 expression (96, 100). In this manner, metformin promotes up-regulation of miRs and performs its anti-tumor effects [96].

## Hypo pharyngeal cancer

### MiR-21-5p

MiR-21-5p, a circulating miR, is significantly up-regulated in hypo pharyngeal carcinoma. A study concluded that metformin prevents cell proliferation in hypo pharyngeal carcinoma possibly via down-regulation of miR-21-5P and up-regulation of programmed cell death protein 4 (PDCD4) [5, 101, 102]. PDCD4 is considered as a tumor suppressor and is correlated with apoptosis in hypo pharyngeal carcinoma cells, which has an inverse correlation with miR-21 expression (103). Studies have demonstrated that the expression of PDCD4 is reduced in several types of cancer cell lines in which miR-21 is over-expressed [104, 105]. The expression of signal transducer and activator of transcription 3 (STAT3) is activated by miR-21 in EMT. Since, STAT3 is enhanced in PDCD4 knockdown human laryngeal carcinoma cell line, miR-21 may be the downstream molecule of PDCD4 and metformin may inhibit miR-21 through up-regulation of PDCD4 [106].

## Non-small-cell lung cancer

### MiR-7

Metformin promotes cell apoptosis and inhibits non-small cell lung cancer cells growth by stimulating the expression of miR-7 through AMPK dependent mechanism [107]. Several studies have shown that over-expression of miR-7 markedly inhibits growth, migration, and invasion of lung cancer cells, by targeting BCL-2, an anti-apoptotic protein which has the ability to suppress tumorigenicity [108, 109]. Up-regulation of miR-7 could dramatically arrest the three signaling pathways of AKT/mTOR, MAPK/Erk, and NF-κB that are involved in the formation, growth, apoptosis, invasion, metastasis, and angiogenesis in lung cancer [107]. Based on studies, circulating miR-7 can be considered as a diagnostic biomarker in patients with non-small-cell lung cancer [110, 111].

### MiR-381

A new study has shed light on how metformin acts in preventing tumorigenesis and metastasis in non-small cell lung cancer. MiR-381 is a new cellular/tissue miR candidate that is probably involved in non-small cell lung cancer. This miR is reported to be a tumor suppressor and a participant in the miR-381-YAP-Snail signaling axis. Metformin suppresses cell proliferation, invasion, and migration in non-small cell lung cancer cells by regulating this pathway [112]. A previous study suggested that metformin may be able to inhibit the function of Yes kinase-associated protein (YAP) in non-small cell lung cancer by preventing the binding of interferon regulatory factor 1 (IRF-1) and YAP promoter [113]. Yap has a transcriptional activation domain that regulates transcription via occupation of the promotor region of the target genes, thus its inhibition leads to suppression of cell proliferation and progression of apoptosis, thereby limiting tumor size [114]. On the other hand, YAP is able to positively influence snail expression, an important factor in induction of EMT. Snail inhibition leads to E-cadherin up-regulation and disruption of EMT in lung cancer cells. Due to the up-regulation of miR-381 by metformin treatment, the expression of YAP and snail are decreased and EMT is inhibited [112].

### MiR-107

Metformin can regulate T cell functions in patients with non-small cell lung cancer by reprograming the differentiation of memory stem T (Tscm) and central memory T (Tcm) which are subsets of memory CD8 + T cells. According to a study by Zhang et al., AMPK activation leads to down-regulation of miR-107 which targets Eomesodermin (Eomes). Eomes is an impressive factor in T cell differentiation. Metformin promotes differentiation of T cells and inhibits their proliferation and expression of programmed cell death protein (PD-1). PD-1 is an inhibitory receptor which is expressed in exhausted T cells during cancer [115]. On the other hand, mTOR which is negatively regulated by AMPK activation can reduce Eomes and increase PD-1 expression (116, 117). Moreover, miR-107 can inhibit tumor growth and metastasis through targeting brain-derived neurotrophic factor (BDNF)/PI3K/AKT axis in human non-small lung cancer [118]. Hence, metformin may lead to affecting expressions of miR-107 via an AMPK-dependent mechanism.

## Osteosarcoma

### MiR-570-3p

The results obtained from a miR microarray assay in osteosarcoma cells showed that metformin causes demethylation of DNA at the CpG islands of miR-570-3p promoter regions, thus elevating the expression of miR-570-3p, a tumor suppressor miR [119]. MiR-570-3p plays an anti-cancer role in various tumors via inhibiting cell proliferation, migration, and invasion [120, 121]. In tissues of osteosarcoma patients, increased expression of miR-570-3p could result in the repression of its target genes translation such as lung cancer metastasis-related protein (LCMR1) and autophagy-related gene 12 (AtG12) which are involved in metastasis and autophagy, respectively, and prevent apoptosis by its ability to directly bind to 3′UTR regions [119]. The role of metformin in over-expressing miR-570-3p highlights its potential role in attenuating metastasis and autophagy by means of miR-570-3p.

## Ovarian cancer

### Let-7

Decreased circulating let-7 expression is an independent prognostic biomarker for ovarian cancer [122]. Metformin suppresses cell migration and invasion by increasing let-7 levels as observed in endometrial cancer [47]. It is assumed that metformin affects let-7 expression by various mechanisms affecting H19/let-7 axis, Lin28/let-7 axis and AMPK phosphorylation. Metformin also acts in a DICER1‐dependent manner and applies its anti-tumor effects [47, 73–76]. The related mechanism is described previously.

### MiR-3127-5p

Resistance to paclitaxel is one of the most prominent causes of failure to chemotherapy in ovarian cancer. Metformin can enhance paclitaxel sensitivity by regulating the expression levels of lncRNA small nucleolar RNA host gene 7 (SNHG7) and miR-3127-5p and impeding the autophagy process in animal models of ovarian cancer and cancer cells. Metformin treatment could abrogate SNHG7 expression. Inhibition of SNHG7 results in overexpression of miR-3127-5p that is considered a direct target of SNHG7 [123]. It is noteworthy that miR-3127-5p, a tissue/cellular miR, acts as an autophagy regulator by activating of PI3K/AKT signaling pathway [124]. As a result of miR-3127-5p elevation, a decrease in the protein levels of microtubule -associated proteins 1A/1B light chain 3 -II (LC3II) and Beclin1, as known autophagy markers, as well as an increase in poly (ADP-ribose) polymerase (PARP) protein were observed [123]. LC3-II is a phosphatidylethanolamine conjugate form of LC3 that is associated with autophagosome maturation [48]. Besides, LC3 reduction in miR‐3127‐5p knocked cells may represent LC3 as a predicted target of miR‐3127‐5p [125]. These data suggest that metformin may influence drug sensitivity in ovarian cancer by attenuating autophagy through SNHG7/miR-3127-5p axis [123].

## Pancreatic cancer

### MiR-150

Role of metformin in inhibition of cell proliferation in pancreatic cancer cells is assumed to be through up-regulation of miR-150 [73]. MiR-150 is identified as a circulating miR and a tumor suppressor in many cancers. In patients with pancreatic cancer, decreased blood levels of miR-150 has been observed [126]. MiR-150 inhibits growth and malignant behavior of cancer cells by binding to 3′UTR of Mucin 4 (MUC4), a high molecular mass proteoglycan, and down-regulates its expression [127, 128]. In pancreatic cancer cells, miR-150 induces its anti-apoptotic effect by reducing c-Myb expression [129]. As a result of c-Myb depletion, its binding to the insulin-like growth factor 1 receptor (IGF-1R) and Bcl-2 promoter decreases. On the other hand transcription and expression of these proteins are inhibited [130]. c-Myb, IGF-1R and Bcl-2 that are over-expressed in pancreatic cancer cells have a controlling role in cell proliferation, apoptosis, and differentiation [129, 131]. Combined treatment of metformin with aspirin suppresses Bcl-2 expression leading to induction of apoptosis in pancreatic cancer cells [132]. On another hand, metformin disrupts interaction between insulin/IGF-1R and G-protein-coupled receptor (GPCR) signaling pathways that have a critical role in the development of pancreatic cancer [133, 134].

### MiR-210-5p

Results of recent studies in human pancreatic cancer cells provide some evidence that the inhibitory effect of metformin on cell viability and expression level of miR-210-5p is dependent on glucose state. Metformin induces a more pronounced expression of miR-210-5p in low glucose conditions. Over-expression of miR-210-5p could lead to inhibition of cell activity that is mediated by down-regulation of 6-phosphofructo-2-kinase/fructose-2,6-bisphosphatase 2 (PFKFB2), which is characterized as a potential target gene of miR-210-5p. Besides, following the repression of PFKFB2, the glycolysis pathway is inhibited. The activity of two key enzymes in the downstream pathway of glycolysis, phosphofructokinase-1 (PFK1) and lactic acid dehydrogenase is also markedly decreased [135].Thus, it has been suggested that blockade of anaerobic glycolysis could be one of the potential anti-cancer mechanisms of metformin. Due to severe reduction of adenosine triphosphate (ATP) production, cancer cells become more susceptive to metformin-induced stress. Consequently, metformin may be more helpful in non-diabetic patients with pancreatic cancer [136].

### MiR-221

MiR-221 is another known oncomiR which is suppressed by metformin in some cancer cell lines such as human pancreatic cancer cell, human cholangiocarcinoma and endothelial progenitor cells. As a result of this inhibition, termination of the cell cycle in G1 phase occurs [137]. Up-regulation of miR-221 has a major role in proliferation and cell cycle processes. Increased levels of miR-221 have been observed in plasma of patients diagnosed with pancreatic cancer [138, 139].

p27, the direct target of miR-221, is a cell-cycle inhibitor which binds to CDK complexes and leads to G1-phase arrest in cancer cells which is negatively modulated by miR-221 [140]. Moreover, p27 expression is increased as a consequence of miR-221 inhibition by metformin in pancreatic cancer cell [141]. Metformin also increases p27 expression through AMPK phosphorylation and causesinhibition of angiogenesis [137]. The reductive and increasing effects of metformin on miR-221 and p27 expression, respectively have also been tangible in studies in diabetic patients [142]. MiR-1 inhibits autophagy by affecting two different pathways: first, by modulating the p27/CDK2/mTOR axis, secondly, by targeting PTEN, AKT and mTOR signaling axis [143, 144]. Mechanism of metformin in pancreatic cancer may be similar to that in animal models of CCl4-treated hepatocellular carcinoma. Metformin causes down-regulation of miR-221, interfering with AKT expression and its downstream effectors such as S6 and eukaryotic translation initiation factor 4E-binding protein 1 (4EBP1) [145].

### MiR-200a

MiR-200a is known as a tumor suppressor and a plasma/serum detectable miR. Some studies have shown that over-expression of miR-200a may repress cell proliferation, cell cycle progression and invasion in pancreatic cancer [146–148]. Result of miR microarray assay in pancreatic tumor cells showed that metformin exhibits anticancer effects by down-regulating miR-200a. However the exact mechanism of metformin in this regard is still obscure [149].

## Prostate cancer

### Mir-708

MiR-708-5p is a circulating miR which acts as a tumor-suppressor by targeting an endoplasmic reticulum (ER) protein neuronatin (NNAT), causing a decrease in intracellular calcium. Metformin treatment dramatically up-regulates miR-708-5p expression resulting in inhibition of calcium uptake by ER. Decline in intracellular calcium causes ER stress and as a result, cell apoptosis is activated. Thus, NNAT is identified as a novel target of metformin in induction of apoptosis for prostate cancer cells [150–153].

## Renal cell carcinoma

### MiR-21

Many studies have confirmed the involvement of miR-21 in carcinogenesis in kidney cells and targeted PTEN [154, 155]. The results of a study examining the effect of metformin on renal cell carcinoma originated cells demonstrated the anti-proliferative properties of metformin via miR-21/PTEN/Akt signaling pathway in an AMPK dependent manner in inhibiting cell proliferation [155]. According to a study by Bera et al., regarding the role of miR-21 and its downstream mechanisms, inhibition of PTEN by miR-21 mediates the Akt- inhibitor of nuclear factor kappa-B kinase subunit beta (IKKβ) axis and leads to NFκB activation. As a result of NFκB activation, the expression of cyclin D1 and CDK4 are increased as observed in renal cancer patients [156, 157]. Increased cyclin D1 levels initiates cell cycle progression and cell proliferation by stimulation of CDK4/6 in renal cells [157, 158].

### MiR-34a

A study performed on human renal cancer cell line treated with metformin, indicated that metformin may induce cell cycle arrest by up-regulating the level of miR-34a, decreasing the level of cyclin D1 expression and enhancing cyclin-dependent kinase inhibitor 1B (p27Kip1) expression [159]. MiR-34a is considered as a tumor-suppressive miR for its suppressing effects on cell cycle, cell invasion, and cell migration in renal cell carcinoma [160]. MiR-34a has anti-proliferative potentials causing down-regulation of several proliferative proteins including cyclin D1, cyclin E, CDK4 and CDK6 which induce cell cycle arrest in G0/G1 phase [161, 162]. Low P27 expression, a CDKs regulator which negatively controls cell cycle in transition of G1 to S phase by inhibiting cyclin E/CDK2 complex, is correlated with the expression of cyclin D1 in patients with renal cell carcinoma [163]. Decreased cyclin D1 levels leads to the release of p27 and induces cell cycle arrest. Metformin has previously shown its antitumor effect by reducing the regulation of cyclin D1 in AMPK-dependent mechanisms [164, 165].

## Skin cancer

### MiR-21

A study on skin keratinocyte cell line viability, suggested the involvement of miR-21/PTEN/Akt signaling pathway in anti-proliferative properties of metformin. Treatment of these cells with metformin leads to significant down-regulation of miR-21 and up-regulation of PTEN which negatively modulate the AKT signaling pathway [166]. On the other hand, studies indicate a positive correlation between the expression of miR-21 and TGF-β. MiR-21 exerts its effects on cell proliferation, migration and angiogenesis in keratinocytes as a downstream molecule of TGF-β by activating TGF-β signaling and targeting PTEN [167, 168]. TGF-β 1 is involved in the progression of many diseases, including cancers [169]. Moreover, Metformin down-regulates miR-21 through antagonizing TGF-β 1 signaling by targeting mothers against decapentaplegic homolog 7 (SMAD7) protein and PTEN in an AMPK independent manner [91, 92]. In fact, metformin induces inhibitory effects on TGF-β-induced AKT-, SMAD- and ERK-dependent phosphorylation signaling pathways by down-regulation of miR-21[170].

### MiR-192-5 and miR-584-3

Metformin up-regulates two other circulating miRs, miR-192-5p and miR-584-3p which function as tumor suppressors directly by binding to 3′UTR region of epidermal growth factor [171–173] containing fibulin-like extracellular matrix protein 1 (EFEMP1) and secretory carrier membrane proteins (SCAMP3), respectively, which play important roles in cell growth and motility. Tseng et al., have demonstrated that knocking EFEMP1 and SCAMP3 down, leads to cell cycle arrest in G2/M phase in human melanoma cell lines. On the other hand, cell invasion and migration were also affected by knocking EFEMP1 down [174–177].

## Conclusion

In recent years, the role of miRs in diagnosis, prognosis and treatment of cancer has been greatly investigated and has opened a new avenue to understand distinct perspectives of cellular processes. Depending on their targets, miRs may have oncogenic or tumor-suppressive effects. Since metformin influences proliferation of cancer cells by modifying dysregulated miRs, their study may assist in elucidating the anticancer properties and mechanisms of metformin.

In this review, we have focused on the therapeutic role of metformin targeting miRs in different types of cancer. Studies investigating the role of metformin in different cancers may help clarify the link between metformin and downstream signaling pathways of related miRs. This may lead to finding new roles for metformin and related miRs as therapeutic molecules in curing cancer in the future.

Considering the safe profile of metformin and its low cost along with its anticancer properties involving regulation of various miRs in different types of cancers, metformin can be proposed as a novel therapeutic adjuvant to conventional chemotherapy regimens with the intention of enhancing outcome.

## Data Availability

Not applicable.
